# 

**Published:** 1916-03

**Authors:** 


### APRIL ISSUE

Vincent's Angina.
DR. RAY KARCHMER DAILY, Houston.

Difficulties in Breast Feeding.
DR. MARY CLEVELAND HARPER, San Antonio.

The Value of Pure Milk Supply and How to Obtain It.
DR. ETHEL LYON HEARD, Galveston.

Surgical Pathology.
DR. VIOLET H. KEILLER, Galveston.

The Examination of Blood Smears as a Diagnostic Asset.
DR. MARTHA A. WOOD, Houston.

### AN IMPORTANT ANNOUNCEMEMT

It gives us a great deal of pleasure to announce that beginning in an early issue of the Texas Medical Journal, there will be a series of articles from the pen of Dr. F. S. White of San Antonio, in which he will deal with various phases of incipient insanity, which are not easily recognized by those who are not expert neurologists. These articles will seek to help all physicians to make an early diagnosis of such cases.

Dr. White has devoted much time to the study of nervous and mental diseases and his years and years of practical, conscientious work as Superintendent of the Insane Asylums at Austin and San Antonio have won for him the recognition of "insanity expert."

Some of the subjects with which this series of articles will deal are as follows:

"Nervous Women and Gynecology."

"Dementia Precox."

"Some Suggestions as to the Prevention of Hereditary Tendencies."

"The Manic Depressive Group of Mental Diseases."

You can not afford to miss this highly instructive series.

### Deaths.

Dr. J. R. McFadden, a pioneer physician of Ellis county, died at his home at Milford, February 6th. Dr. McFadden was one of the oldest physicians in Ellis county.

Dr. H. V. Collins, of Jacksonville, died February 12th.

Dr. F. U. Meadows, a prominent dentist of Greenville, died suddenly at his home, 1613 North Wesley Street, on February 8th, from a stroke of apoplexy.

Dr. C. W. Westmoreland, formerly of Laredo, died at his old home in Columbus, Miss., on January 15th.

Dr. Lois Fitch Mansfield, believed to be the oldest woman physician in the United States, died at her home in Santa Barbara, Cal.,

January 19th. She is said to have conducted the first open air school in America.

Dr. J. T. Avirett, of Taylor, died January 27th.

Dr. W. H. Parks died very suddenly at his home near Cleburne January 23d.

Dr. J. D. Burditt, a prominent specialist of Houston, was found dead in the bath tub January 24th. He was subject to heart trouble, and it is supposed that his death was due to heart failure.

Dr. Fletcher, of Dodsonville, died February 1st, as a result of an automobile wreck the day before

Dr. Daniel W. Snow, of Campbell, died February 16th.

Dr. W. L. Baird died at Huntsville February 17th. The body was taken to Maysfield, his old home, for interment.

Dr. Albert H. Foster died at the home of his parents in Marlin February 12th.

Dr. D. F. Crawford, of Amis, died at his home February 23d.

Dr. M. L. Hyden, of Boerne, died February 21st.

Dr. J. M. Nicks, of Bryan, died February 18th.

